# Innovative Strategies to Identify *M. tuberculosis* Antigens and Epitopes Using Genome-Wide Analyses

**DOI:** 10.3389/fimmu.2014.00256

**Published:** 2014-06-25

**Authors:** Annemieke Geluk, Krista E. van Meijgaarden, Simone A. Joosten, Susanna Commandeur, Tom H. M. Ottenhoff

**Affiliations:** ^1^Department of Infectious Diseases, Leiden University Medical Center, Leiden, Netherlands

**Keywords:** cellular immunity, CD4, CD8, *Mycobacterium tuberculosis*, TB, T-cell epitopes, vaccines

## Abstract

In view of the fact that only a small part of the Mtb expressome has been explored for identification of antigens capable of activating human T-cell responses, which is critically required for the design of better TB vaccination strategies, more emphasis should be placed on innovative ways to discover new Mtb antigens and explore their function at the several stages of infection. Better protective antigens for TB-vaccines are urgently needed, also in view of the disappointing results of the MVA85 vaccine, which failed to induce additional protection in BCG-vaccinated infants (1). Moreover, immune responses to relevant antigens may be useful to identify TB-specific biomarker signatures. Here, we describe the potency of novel tools and strategies to reveal such Mtb antigens. Using proteins specific for different Mtb infection phases, many new antigens of the latency-associated Mtb DosR-regulon as well as resuscitation promoting factor proteins, associated with resuscitating TB, were discovered that were recognized by CD4^+^ and CD8^+^ T-cells. Furthermore, by employing MHC binding algorithms and bioinformatics combined with high-throughput human T-cell screens and tetramers, HLA-class Ia restricted polyfunctional CD8^+^ T-cells were identified in TB patients. Comparable methods, led to the identification of HLA-E-restricted Mtb epitopes recognized by CD8^+^ T-cells. A genome-wide unbiased antigen discovery approach was applied to analyze the in vivo Mtb gene expression profiles in the lungs of mice, resulting in the identification of IVE-TB antigens, which are expressed during infection in the lung, the main target organ of Mtb. IVE-TB antigens induce strong T-cell responses in long-term latently Mtb infected individuals, and represent an interesting new group of TB antigens for vaccination. In summary, new tools have helped expand our view on the Mtb antigenome involved in human cellular immunity and provided new candidates for TB vaccination.

## Introduction

### *M. tuberculosis* antigen discovery: Traditional approaches

Antigen discovery efforts have been a core component of mycobacterial research for over several decades, and have been markedly facilitated since the availability of the *Mycobacterium tuberculosis* (*Mtb*) genome sequence (2). A lot of different antigen discovery approaches have been described, among which are biochemical-, genetic-, expression library-, and peptide T-cell epitope-based approaches. Using animal models, antigens with protective potential against *Mtb* infection have been discovered, some of which have moved into human clinical trials (phase 1/2a) (3). Nevertheless, T-cell epitopes have been identified in only 7% of all predicted 4000 open reading frames (ORFs) of *Mtb*, and the top 30 most frequently studied protein antigens contain 65% of the known epitopes (4). This leaves the *Mtb* “antigenome” incompletely identified, which may be especially relevant in relation to *Mtb’s* phase-dependent variation in gene expression (see below) in response to varying environmental factors (5, 6).

Selection of vaccine candidates has been based largely on empirical observations in the mouse, guinea pig, and non-human primate *Mtb* infection models and only a few antigens (mostly those secreted during active replication of *Mtb*) have been exploited as human vaccine candidates (7). Since *Mtb* alters its gene expression profile significantly during intracellular stress inside host macrophages, its antigen repertoire which is expressed and exposed to the immune system varies considerably during different stages of infection under the pressure of various human host defense mechanisms. A more profound understanding of the actual *Mtb* antigenome expressed during different phases of *Mtb* infection, particularly in the lung, the main target organ of *Mtb* and the identification of the major T-cell epitopes involved, is key to the design of better TB-vaccines and TB correlates of immunity.

Almost all TB-vaccine antigen discovery approaches have implicitly relied on the assumption that the *Mtb* antigens studied are expressed and presented by infected cells, where they are supposedly recognized by T-cells that execute an appropriate effector response. The latter either assist phagocytes in controlling or eliminating live bacteria through various intracellular pathways (phagosomal maturation and phagolysosomal fusion; oxidative/nitrate intermediates; oxygen/nutrient deprivation; the activity of defensins and other anti-microbial peptides and enzymes; autophagy; apoptosis) (8), or – alternatively direct killing of infected cells. Despite the significant advances made recently, relatively little is known about the *Mtb* antigen repertoire, which is truly expressed by the tubercle bacillus during its infection cycle in human cells. Although abundantly expressed proteins of *Mtb* are often the primary targets of research, less prominently expressed antigens may have equally good or even superior vaccine potential. Better insight into the antigen repertoire available for immune recognition on infected cells, its dynamic changes as well as the quantitative relationship between the various antigens expressed, should provide new directions for antigen discovery and vaccine testing, with the potential to complement or change current strategies used in TB-vaccine-design.

### Classical *Mtb* antigen discovery methods and the risk of tunnel vision

Indirect discovery approaches have mostly been the basis for currently available evidence supporting the recognition of *Mtb* antigens, including those on infected cells. Whereas the protective potential of *Mtb* antigens is typically demonstrated using effective vaccine platforms in animal models, the selection of the antigens to be tested in such platforms is often biased and limited by the antigen discovery procedures used. For example, as outlined above, many studies have concentrated on antigens that are highly expressed by bacteria under *in vitro* culture conditions in liquid growth media. However, it is improbable that the same bacterial transcriptomic or proteomic profiles expressed under optimal laboratory growth conditions are the same as those expressed during *in vivo* host infection.

A further important bias is that most *Mtb* antigens recognized by human cells have been identified using IFN-γ assays as read outs. As mentioned above, a considerable number of antigens eliciting CD4^+^ IFN-γ Th1-cell responses has been identified (9), but this represents almost certainly only a fraction of the potential *Mtb* “antigenome.” The number of IFN-γ-releasing antigen-specific T-cells and the amount of total IFN-γ released have remained widely used surrogate markers for the pro-inflammatory immune response against *Mtb*. Thus, the antigens activating other immune cells, including CD4^+^ T-cells that produce other cytokines than IFN-γ such as Th2 cells, Tregs, Th17/22, cells and cytolytic cells, as well as non-classically restricted (MHC-Ib, see below) human T-cell subsets, remain largely incomplete or even unknown. However, the lack of (application of) well-developed methods to identify *Mtb*-responsive T-cells other than classical Th1 or proliferative responses have contributed to this bias in *Mtb* antigens identified. Thus, it is vital to develop better and more diverse assays that can be applied to detect *Mtb*-induced responses across all relevant human T-cell compartments.

### Scope of this review

**Figure 1:**
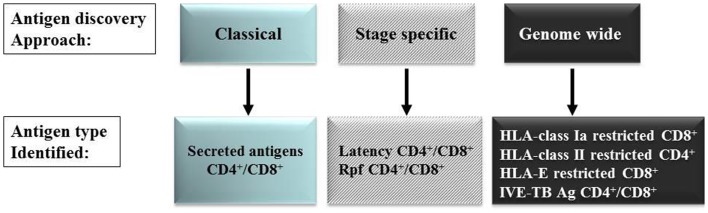
***Mtb* antigen discovery approaches**.

Since our knowledge of the human *Mtb* antigenome is far from complete as most currently known *Mtb* antigens were identified using IFN-γ (Th1) production as read-out, and because there is limited knowledge about the vaccine potential (protective efficacy) of most antigens, we have followed several alternative strategies to discover new *Mtb* antigens. Below, we will review the five approaches we have pursued in recent years (Figure 1). It is likely that many unexplored antigens for classical or non-classical T-cells exist in the *Mtb* antigenome that may possess vaccine potential, but appropriate tools and technologies are required to reveal these. Improved and rational selection is needed to identify candidate antigens with vaccine potential, based on comprehensive knowledge of their patterns of expression, broad immunogenicity, suitability for processing, HLA-binding and induction of protective immunity in relevant model systems.

## New *Mtb* Antigen Discovery Approaches

### Infection stage-specific *Mtb* gene expression analyses

#### *Mtb* DosR-regulon encoded proteins as latency antigens

As mentioned above, we have hypothesized that the design of improved vaccination strategies requires a better understanding of the *Mtb* proteins that are expressed during the different phases of the *Mtb* intracellular life cycle, and their recognition by human T-cell subsets.

During *in vivo Mtb* infection, environmental and host immune factors induce bacterial dormancy, in the course of which *Mtb* enters a state of non-or slowly replicating persistence, decreases its metabolic activity and alters its gene expression pattern (5, 10). This adjustment by the bacterium is thought to enhance its resistance to environmental and host immune stress. Under *in vitro* conditions that are considered to imitate part of the environment encountered by tubercle bacilli *in vivo* in the immunocompetent host (11) such as hypoxia, low dose nitric oxide, CO exposure or in IFN-γ-activated macrophages, *Mtb* induces the expression of the 48 gene encoding DosR (Rv3133c) regulon (12). In view of this infection phase-specific upregulation, we first decided to study T-cell responses to these 48 DosR-regulon encoded antigens (so-called TB latency antigens) in various ethnically and geographically distinct cohorts in Europe (Italy, Germany, The Netherlands) and Africa (The Gambia, Ethiopia, Uganda, and South Africa) (13–19). In all cohorts we observed T-cell recognition of *Mtb* DosR-regulon encoded latency antigens, particularly in TST^+^ individuals [designated latent TB (LTBI)] compared to (ex-) TB patients. Thus, T-cell proliferation and IFN-γ production to *Mtb* DosR-regulon encoded latency antigens are characteristically seen in LTBI in diverse genetic and geographic populations.

An unexpected but important finding was that *Mtb* DosR-regulon encoded antigens were very poorly if at all recognized in previously BCG-vaccinated individuals (20). Additionally, BCG-vaccinated HLA-DR3 and HLA-A2 transgenic mice (21) failed to respond to these antigens. The explanation for this lack of induction of immune responses was not due to BCG’s ability to express the DosR-regulon because transcriptional profiling of 14 different BCG strains, cultured under hypoxia, and nitric oxide exposure *in vitro*, showed that genes of the DosR-regulon (except Rv3133) were expressed to similar extents as observed in *Mtb*. Moreover, comparison of the amino acid sequences of *M. bovis* BCG and *Mtb* showed at least 97% homology with 85% of the genes being completely identical (20). The explanation for this phenomenon thus remains unknown, although it may relate to an inability of BCG to enter a state of latency following intradermal immunization. In agreement with the findings in human LTBI, the *Mtb* DosR-regulon encoded latency antigens Rv1733c, Rv2031c, and Rv2626c, were more prominently recognized in chronic infected mice than in acute phase infection, whereas the secreted protein Ag85B (Rv3804c) showed the opposite phenotype (18), again highlighting the preferential recognition of these antigens in latent or long term as opposed to acute, early phase infection.

An important group of individuals, the study of which shed more light into this matter was composed of individuals who had been *Mtb* infected for over 30 years ago, as witnessed by their positive Mantoux skin tests, yet never developed disease despite not having received any therapeutic treatment. Detailed immune profiling experiments with cells from these long-term infected individuals demonstrated strong *Mtb* DosR-regulon encoded antigen-specific T-cell responses. Various epitopes were identified that induced mono- and polyfunctional CD4^+^ and CD8^+^ T-cell responses, in particular IFN-γ^+^TNF-α^+^ CD8^+^ effector memory- or effector T-cells (22).

Considering the inability of BCG to induce immune responses to latency antigens, TB vaccination strategies started to consider incorporating DosR-regulon encoded antigens as a strategy to complement and improve the current BCG vaccine. This was demonstrated in TB mouse models using rBCG_ureC:hly expressing defined latency-associated antigens and test this construct for long-term protection against an isolate of the *Mtb* Beijing/W lineage (23). Expression of Rv2659c, Rv3407, and Rv1733c by rBCG_ureC:hly showed long-term protection superior to BCG in both lung and spleen compared to rBCG not expressing latency antigens at day 200 post-infection after intradermal vaccination of mice. Even a latency antigen hsp16-derived peptide linked to PAM2Cys adjuvant induced more efficient protection than BCG (24).

Another type of latency antigens is represented by the so-called “starvation” antigens, which are expressed under conditions of nutrient deprivation. A hybrid protein consisting of two secreted, acute phase-specific antigens, Ag85B and ESAT-6, fused to the nutrient stress-induced antigen Rv2660c, was constructed. In pre-exposure mouse models this hybrid protein formulated in CAF01, promoted hybrid-specific T-cells, in particular polyfunctional CD4**^+^** T-cells and more efficient containment of late-stage infection than the Ag85B-ESAT-6 vaccine and BCG (25). Moreover, in latent TB mouse models, post-exposure immunization with this hybrid controlled TB reactivation and significantly lowered the number of bacteria in the lung compared to adjuvant control mice.

Immunization of cynomolgus macaques with the multistage Ag85B-ESAT-6-Rv2660c protein in IC31 adjuvant as a boost to vaccination with BCG delayed and reduced clinical disease after challenge with *M. tuberculosis* and also prevented reactivation of latent infection (26). This boosting regimen resulted in efficient control of *M. tuberculosis* infection and reduced rates of clinical disease. Importantly, it improved survival of the NHP compared to BCG alone and the vaccinated monkeys did not reactivate latent infection after treatment with anti-TNF antibody.

Considering routine BCG vaccination practice in most TB endemic countries, improved, rationally designed *Mtb* subunit vaccines could be employed by simultaneous vaccination with BCG, or booster vaccination on top of BCG, or by constructing improved recombinant BCG strains expressing such antigens.

#### Resuscitation promoting factors as antigens

Although the factors that trigger bacterial reactivation and resumption of intracellular growth remain largely known, *Mtb* resuscitation promoting factors (Rpfs), which are secreted proteins with high homology to the hormone-like protein secreted by *Micrococcus luteus* (*M. luteus*) are believed to play an important role in this respect (27, 28). The *Mtb* genome encodes five such *rpf* genes [*Rv0867c (rpfA)*, *Rv1009 (rpfB)*, *Rv1884c (rpfC)*, *Rv2389c (rpfD)*, and *Rv2450c (rpfE)*] that are able to stimulate the growth of dormant mycobacteria and expression of the five Rpf proteins is observed *in vitro* in actively replicating *Mtb*, in BCG as well as in *Mtb-*infected human tissue (29–31). Addition of Rpf proteins to sputa from TB patients improves the sensitivity of culture based detection of live *Mtb* in certain conditions (32, 33). There is differential *rpf* expression in cultures grown under hypoxia, nutrient starvation, acidic conditions, stationary, non-cultivable, and resuscitation phase-like conditions, suggesting that the role of *Mtb* Rpfs may be infection stage dependent but likely is not identical for all Rpfs (34).

In view of the role of *Mtb* Rpf proteins in the resuscitation of mycobacteria, immunity against these proteins may reflect the ability to detect the presence of actively replicating *Mtb* organisms at an early stage. Thus, immunity against Rpf proteins may play a role in host control bacterial reactivation. In line with the immunogenicity found in mice for Rv0867c, Rv1009, Rv2389c, and Rv2450c (35), we identified the first human *Mtb* Rpf-specific T-cell responses against *Mtb* Rpfs (19), showing IFN-γ production in TST^+^ individuals in response to Rv1009, Rv1884c, and Rv2450c and to a lesser extent Rv0867c, whereas hardly any IFN-γ was detected in individuals without a positive Mantoux. More detailed analyses of the immune responses to *Mtb* Rpf proteins in *Mtb*-exposed individuals, including long-term LTBI non-progressors showed frequent and significant T-cell responses against both Rv0867c and Rv2389c and identified novel *Mtb* Rpf epitopes, including a single highly dominant peptide epitope in Rv2389c. Of note, *Mtb* Rpf-specific polyfunctional memory CD4^+^ and particularly CD8^+^ T-cell memory responses were observed in response to Rv0867c and Rv2389c Rpf proteins. The polyfunctional phenotype of these cells was both single and double cytokine producing CD4^+^ and CD8^+^ T-cells, supporting the concept that CD8^+^ T-cells may be important in long-term control of *Mtb* infection. Based on these collective studies, we envisage that a combination of multiple phase-specific antigens may significantly improve the protective potential of new TB-vaccines (25, 36). Thus, subunit vaccines based on a combination of latency and Rpf antigens could be designed that induce responses able to eliminate *Mtb* bacilli in their dormant state, as well as inhibit *Mtb* bacilli that are trying to recommence active replication.

### Unbiased *Mtb* “genome wide” antigen discovery approaches

#### HLA-class Ia presented human CD8^+^ T-cell epitopes

Despite the fact that CD4^+^ T-cells play a vital role in immunity against *Mtb*, it is becoming evident that also CD8^+^ T-cells contribute to host defense against *Mtb* by virtue of their ability to produce pro-inflammatory cytokines (9, 37, 38), lyse infected host cells (39), and kill mycobacteria (40). Despite the fact that the antigens and epitopes activating human *Mtb*-specific CD8^+^ T-cell responses have been less well defined than those for CD4^+^ T-cells, some groups have been able to isolate such CD8^+^ T-cells from humans (41–44). Unexpectedly, it was reported that *ex vivo* frequencies of CD8^+^ T-cells recognizing epitopes from six different *Mtb* proteins in patients with active TB were lower as evaluated by specific tetramers, but normalized following therapy to frequencies comparable to subjects with LTBI. Additionally, CD8^+^ T-cells with an IL-2^+^/IFN-γ^+^ phenotype were particularly reduced or found absent in active TB patients (45). Nevertheless, it has remained challenging to identify the role and function of CD8^+^ T-cells in TB, urging for new strategies and appropriate tools to decipher their specificities. Using classic approaches it would not be feasible to screen the approximately 1 million possible 9-mers in the *Mtb* proteome for CD8^+^ T-cell responses.

A recently described approach addressed this issue exploiting an integrated computational and proteomic approach to screen 10% of the *Mtb* proteome for antigens that are recognized by CD8^+^ T-cells: using a synthetic *Mtb* peptide library consisting of 15-mers (11 aa overlap) with high probability of containing CD8^+^ T-cell epitopes, IFN-γ release by *Mtb*-specific, HLA-class I-restricted CD8^+^ T-cell clones was measured by ELISPOT assay (46). This study identified the EsxJ family, PE9, and PE_PGRS42 as three novel CD8 antigens and validated the use of peptide library-based approaches a new tool for identification of *Mtb* epitopes recognized by CD8^+^ T-cells.

An alternative method of combined bioinformatics- and functional immunological screening strategies, called “reverse antigen discovery,” was applied by our group to identify HLA-class Ia restricted, CD8^+^ T-cell antigens. Through peptide-binding prediction algorithms, potential peptide epitopes of *Mtb* antigens were identified restricted by three major HLA-class Ia supertypes (HLA-A2, -A3, and -B7) which together cover more than 80% of the population from different ethnic groups (47). Over 400 synthetic *Mtb* peptides with predicted binding affinities for HLA-A*0201, HLA-A*0301, and HLA-B*0702 (representing the above supertypes) were tested for HLA-binding and induction of proliferation of CD8^+^ T-cells. This study led to the identification of >60 new *Mtb* epitopes. Further validation of the most interesting epitopes was executed using HLA-class I-tetramers and assessment of peptide-induced intracellular cytokine staining to measure multifunctional CD8^+^ T-cell responses in cured TB patients and healthy control individuals. In depth analysis of 18 prominently recognized HLA-A*0201-binding, *Mtb* peptides using CD8^+^ T-cells of cured TB patients, showed IFN-γ, IL-2, and TNF-α, mono-, dual-, and triple-positive CD8^+^ T-cells. Interestingly, in *Mtb*-non-infected individuals these polyfunctional CD8^+^ T-cells were absent, which argues for their priming during *in vivo Mtb* infection. Thus, this study is consistent with the notion that there is a much broader repertoire of CD8^+^ T-cells, which can be identified with specific bioinformatic approaches combined with functional immune assays.

An additional alternative strategy, not yet applied in the identification of new *Mtb* antigens, could be the use of HLA-conditional ligands for high-throughput tetramer generation (48). This technique allows production of HLA ligands that form stable complexes with HLA molecules but can be cleaved upon UV irradiation. The resulting empty, peptide-receptive HLA molecules can be loaded under native conditions with selected epitopes in a high-throughput and HLA-epitope tetramers can be generated and subsequently used for T-cell detection.

Summarized innovative detection methods for CD8^+^ T-cells can and will allow identification of protective- and pathogenic immunity, which can be used to monitor vaccine- and treatment efficacy.

#### HLA-class II presented^+^ human T-cell epitopes

A similar genome-wide approach was recently followed by Sette et al. to identify CD4^+^ T-cell epitopes: the *Mtb* genome was mined for potential peptide epitopes presented by HLA-class II molecules to CD4^+^ T-cells (49). The approach relied on predictions of HLA-binding capacity for a panel of DR, DP, and DQ alleles representative of those most commonly expressed in the general population, coupled with high-throughput ELISPOT assays. They found that secreted antigens as well as proteins involved in the active secretion process were dominant targets of the CD4^+^ T-cell response in the latently infected individuals tested in San Diego who successfully contain *Mtb* infection.

Responses were highly focused on three broadly immunodominant antigenic islands, all related to bacterial secretion systems and composed by several distinct ORFs. These data suggest that vaccination with one or few defined antigens will fail to replicate the response associated with natural immunity. Importantly, they also found that the CD4^+^ T-cells responding were largely restricted to the CXCR3^+^CCR6^+^ memory subset. The identification of this immunodominant population of memory T-cells characterized suggests that the response is shaped uniquely by *Mtb*-associated factors.

In analogy to the above results for CD8^+^ T-cells, these results again underline the power of an unbiased, genome-wide, *Mtb* antigen discovery approaches.

#### HLA-class Ib presented CD8^+^ human T-cell epitopes

The identification of *Mtb* antigens for human CD8^+^ T-cells focus has been focused almost entirely on classical HLA-class Ia and to a lesser extent, on CD1 a, b, c restricted CD8^+^ T-cell responses (50). In this respect, the polymorphism of the classical HLA-class Ia molecules (HLA-A, -B, -C) which include more than 500, 850, or 270 unique alleles, respectively, is an important factor to be considered for vaccine development due to the considerable variations in peptides that can bind to each HLA-class Ia molecule. In contrast, the HLA-class Ib genes HLA-E, -F, and -G exhibit restricted polymorphism with only 3, 4, and 10 alleles, respectively (51). This might argue for distinct roles for MHC-class Ia and class Ib molecules in host defense to infectious diseases. Non-classical HLA-class I molecules can present antigens of both self and foreign (microbial) origin to CD8^+^ T-cells (52–54). The fact that their allelic variation is limited provides an opportunity for vaccine-design. Since the two most important variants of HLA-E, HLA-E^R^ (E*0101), and HLA-E^G^ (E*0103), occur in equal frequencies amongst different populations (55) and can present antigens derived from pathogens including *Mtb, Mtb* antigen(s) recognized by HLA-E restricted CD8^+^ T-cell clones represent interesting targets for vaccine development. Moreover, HLA-E is not downregulated by HIV in contrast to HLA-A and -B offering possibilities for vaccination of HIV-infected individuals as well (56). However, the nature of the epitopes recognized by HLA-E restricted CD8^+^ T-cells remained unknown until recently (57).

Although HLA-E molecules can clearly interact with CD94 molecular complexes expressed predominantly by NK cells, HLA-E is also known to trigger microbial-specific cytotoxic CD8^+^ T-cells (58, 59). In mice, CD8^+^ T-cells restricted by the murine HLA-E equivalent, Qa-1 were found to have the ability to induce immune-suppression (60), revealing yet another function of non-classically restricted CD8^+^ T-cells.

To identify HLA-E-restricted *Mtb* antigens which could be exploited for TB vaccination, we applied bioinformatics, HLA-E peptide-binding assays, and immunological screening, in analogy to the approach used for identification of classical MHC-Ia restricted CD8 T-cell epitopes (61). With this method we identified 69 *Mtb* peptides, derived from a large variety of *Mtb* antigens, which were presented by HLA-E molecules to human CD8^+^ T-cells. We could demonstrate that CD8^+^ T-cells from both mycobacterium responsive adults and BCG-vaccinated infants, but not negative controls, proliferated in response to the identified *Mtb* peptides. These CD8^+^ T-cells displayed cytotoxic activity against target-cells expressing HLA-E loaded with specific peptides, in the absence of any class Ia molecules, and were able to lyse *M. bovis* BCG infected human macrophages demonstrating that HLA-E restricted antigens are naturally processed during infection. Besides, several HLA-E-restricted, *Mtb*-specific CD8^+^ T-cells were able to suppress proliferation of bystander CD4^+^ T-cells. This suppression depended on cell–cell contact and was mediated, at least in part, by membrane bound TGFβ1. These data underscore that human CD8^+^ T-cells are highly multifunctional, and can combine diverse functions such as suppressive and cytotoxic functions. If BCG vaccination would be able to prime HLA-E restricted T-cell responses, HLA-E peptide-based vaccines might be able to boost such BCG-primed responses. The dual character of the response induced in the context of HLA-E may lead to induction of protection as well as balanced regulation of inflammation, which, might be exploited to limit inflammatory pathology in TB.

#### *In vivo* expressed *Mtb* (IVE-TB) antigen-derived human T-cell epitopes

Since the lung represents the principal organ where TB disease is manifested, vaccine-induced immune responses need to target *Mtb* during pulmonary infection. In this respect, it is important to note the significant impact of the alteration of *Mtb*’s gene expression profile during intracellular stress inside host alveolar macrophages, on the *Mtb* antigen repertoire that is presented to the immune system. Thus, we hypothesized that a more profound understanding of the real time *Mtb* “antigenome” expressed during infection in the lung and its recognition by the human immune system, including non-classical T- and B-cells, is vital to develop better vaccination strategies. To identify novel antigens with vaccine potential, we used an unbiased genome-wide approach based on comprehensive gene expression data from *Mtb* during infection of four genetically related but distinct mouse strains (62). These strains represent key features of human TB with a spectrum of TB susceptibility, including the development of necrotic lesions and granuloma formation, which are regulated by the *super-susceptibility to tuberculosis 1* (*sst1*) locus as well as modifier background genes (62). We investigated the *in vivo* expression of 2170 *Mtb* genes, most of which represent the first gene of each predicted *Mtb* operon, during infection in the lungs. To select candidate antigens, stringent selection approaches were then applied to identify a list of *in vivo* expressed *Mtb* (designated IVE-TB) genes. The resulting 16 most consistently expressed *Mtb* genes were produced as recombinant proteins and their immunogenicity was analyzed in PBMC of TST^+^ healthy, TB affected individuals, TB patients as well as long-term LTBI. Seven of the identified IVE-TB antigens were strongly immunogenic in TST^+^, ESAT-6/CFP10-responsive individuals, but not in E/C negative TST^+^ individuals and healthy mycobacterial naïve individuals, indicating that these antigens are presented during natural *Mtb* infection. Importantly, IVE-TB antigen-specific responses could be detected in long-term LTBI, who had been exposed to *Mtb* many years ago yet never developed TB symptoms despite not having had preventive treatment. The most pronounced T-cell subsets recognizing IVE-TB antigens were identified as IFN-γ^+^/TNF-α^+^ CD8^+^ T-cells and TNF-α^+^/IL-2^+^ CD154^+^CD4^+^ T-cells of which the former were major contributors to IFN-γ production. Since IFN-γ^+^/TNF-α^+^ CD8^+^ T-cells were also the most prominent subset in the response to Rpf and DosR proteins, this suggests that the development of specific differential T-cell subsets may be unrelated to the nature of the specific protein antigen involved.

Thus, the analysis of *in vivo* expression patterns during pulmonary *Mtb* infection to identify IVE-TB antigens, combined with detailed immune profiling in humans, led to the identification of IVE-TB as a new class of TB antigens, with the potential for TB vaccination. Our most recent results indeed reveal their protective efficacy in various animal models (63). Importantly, IVE-TB antigen discovery strategies can be applied also to other infectious diseases caused by complex pathogens and represent a novel approach for antigen discovery in general.

## Conflict of Interest Statement

The authors declare that the research was conducted in the absence of any commercial or financial relationships that could be construed as a potential conflict of interest.

## References

[1] Tameris MD Hatherill M Landry BS Scriba TJ Snowden MA Lockhart S Safety and efficacy of MVA85A, a new tuberculosis vaccine, in infants previously vaccinated with BCG: a randomised, placebo-controlled phase 2b trial. Lancet (2013) 381(9871):1021–8 10.1016/S0140-6736(13)60177-4 23391465 PMC5424647

[2] Cole ST Brosch R Parkhill J Garnier T Churcher C Harris D Deciphering the biology of *Mycobacterium tuberculosis* from the complete genome sequence. Nature (1998) 393:537–44 10.1038/31159 9634230

[3] Ottenhoff TH Doherty TM van Dissel JT Bang P Lingnau K Kromann I First in humans: a new molecularly defined vaccine shows excellent safety and strong induction of long-lived *Mycobacterium tuberculosis*-specific Th1-cell like responses. Hum Vaccin (2010) 6:1007–15 10.4161/hv.6.12.13143 21178394

[4] Blythe MJ Zhang Q Vaughan K de Castro R Jr Salimi N Bui HH An analysis of the epitope knowledge related to mycobacteria. Immunome Res (2007) 3:10 10.1186/1745-7580-3-10 18081934 PMC2228276

[5] Betts JC Lukey PT Robb LC McAdam RA Duncan K Evaluation of a nutrient starvation model of *Mycobacterium tuberculosis* persistence by gene and protein expression profiling. Mol Microbiol (2002) 43:717–31 10.1046/j.1365-2958.2002.02779.x 11929527

[6] Voskuil MI Schnappinger D Visconti KC Harrell MI Dolganov GM Sherman DR Inhibition of respiration by nitric oxide induces a *Mycobacterium tuberculosis* dormancy program. J Exp Med (2003) 198:705–13 10.1084/jem.20030205 12953092 PMC2194188

[7] Ottenhoff TH Kaufmann SH Vaccines against tuberculosis: where are we and where do we need to go? PLoS Pathog (2012) 8:e1002607 10.1371/journal.ppat.1002607 22589713 PMC3349743

[8] Ottenhoff TH New pathways of protective and pathological host defense to mycobacteria. Trends Microbiol (2012) 20:419–28 10.1016/j.tim.2012.06.002 22784857

[9] Ottenhoff TH Lewinsohn DA Lewinsohn DM Human CD4 and CD8 T cell responses to *Mycobacterium tuberculosis*: antigen specificity, function, implications and applications. In: Kaufmann SH Britton WJ, editors. Handbook of Tuberculosis. Weinheim: Wiley-VCH Verlag GmbH & Co. KGaA (2008). p. 119–156

[10] Wayne LG Hayes LG An in vitro model for sequential study of shiftdown of *Mycobacterium tuberculosis* through two stages of nonreplicating persistence. Infect Immun (1996) 64:2062–9 8675308 10.1128/iai.64.6.2062-2069.1996 PMC174037

[11] Shi L Jung YJ Tyagi S Gennaro ML North RJ Expression of Th1-mediated immunity in mouse lungs induces a *Mycobacterium tuberculosis* transcription pattern characteristic of nonreplicating persistence. Proc Natl Acad Sci U S A (2003) 100:241–6 10.1073/pnas.0136863100 12506197 PMC140939

[12] Schnappinger D Ehrt S Voskuil MI Liu Y Mangan JA Monahan IM Transcriptional adaptation of *Mycobacterium tuberculosis* within macrophages: insights into the phagosomal environment. J Exp Med (2003) 198:693–704 10.1084/jem.20030846 12953091 PMC2194186

[13] Black GF Thiel BA Ota MO Parida SK Adegbola R Boom WH Immunogenicity of novel DosR regulon-encoded candidate antigens of *Mycobacterium tuberculosis* in three high-burden populations in Africa. Clin Vaccine Immunol (2009) 16:1203–12 10.1128/CVI.00111-09 19553548 PMC2725533

[14] Goletti D Butera O Vanini V Lauria FN Lange C Franken KL Response to Rv2628 latency antigen associates with cured tuberculosis and remote infection. Eur Respir J (2010) 36:135–42 10.1183/09031936.00140009 19926735

[15] Leyten EM Lin MY Franken KL Friggen AH Prins C van Meijgaarden KE Human T-cell responses to 25 novel antigens encoded by genes of the dormancy regulon of *Mycobacterium tuberculosis*. Microbes Infect (2006) 8:2052–60 10.1016/j.micinf.2006.03.018 16931093

[16] Lin MY Ottenhoff TH Host-pathogen interactions in latent *Mycobacterium tuberculosis* infection: identification of new targets for tuberculosis intervention. Endocr Metab Immune Disord Drug Targets (2008) 8:15–29 10.2174/187153008783928398 18393920

[17] Lin MY Ottenhoff TH Not to wake a sleeping giant: new insights into host-pathogen interactions identify new targets for vaccination against latent *Mycobacterium tuberculosis* infection. Biol Chem (2008) 389:497–511 10.1515/BC.2008.057 18953716

[18] Roupie V Romano M Zhang L Korf H Lin MY Franken KL Immunogenicity of eight dormancy regulon-encoded proteins of *Mycobacterium tuberculosis* in DNA-vaccinated and tuberculosis-infected mice. Infect Immun (2007) 75:941–9 10.1128/IAI.01137-06 17145953 PMC1828490

[19] Schuck SD Mueller H Kunitz F Neher A Hoffmann H Franken KL Identification of T-cell antigens specific for latent *Mycobacterium tuberculosis* infection. PLoS One (2009) 4:e5590 10.1371/journal.pone.0005590 19440342 PMC2680040

[20] Lin MY Geluk A Smith SG Stewart AL Friggen AH Franken KL Lack of immune responses to *Mycobacterium tuberculosis* DosR regulon proteins following *Mycobacterium bovis* BCG vaccination. Infect Immun (2007) 75:3523–30 10.1128/IAI.01999-06 17502400 PMC1932964

[21] Geluk A Lin MY van Meijgaarden KE Leyten EM Franken KL Ottenhoff TH T-cell recognition of the HspX protein of *Mycobacterium tuberculosis* correlates with latent *M. tuberculosis* infection but not with *M. bovis* BCG vaccination. Infect Immun (2007) 75:2914–21 10.1128/IAI.01990-06 17387166 PMC1932904

[22] Commandeur S Lin MY van Meijgaarden KE Friggen AH Franken KL Drijfhout JW Double- and monofunctional CD4 and CD8 T-cell responses to *Mycobacterium tuberculosis* DosR antigens and peptides in long-term latently infected individuals. Eur J Immunol (2011) 41:2925–36 10.1002/eji.201141602 21728172

[23] Reece ST Nasser-Eddine A Dietrich J Stein M Zedler U Schommer-Leitner S Improved long-term protection against *Mycobacterium tuberculosis* Beijing/W in mice after intra-dermal inoculation of recombinant BCG expressing latency associated antigens. Vaccine (2011) 29:8740–4 10.1016/j.vaccine.2011.07.144 21871515

[24] Gowthaman U Singh V Zeng W Jain S Siddiqui KF Chodisetti SB Promiscuous peptide of 16 kDa antigen linked to Pam2Cys protects against *Mycobacterium tuberculosis* by evoking enduring memory T-cell response. J Infect Dis (2011) 204:1328–38 10.1093/infdis/jir548 21933875

[25] Aagaard C Hoang T Dietrich J Cardona PJ Izzo A Dolganov G A multistage tuberculosis vaccine that confers efficient protection before and after exposure. Nat Med (2011) 17:189–94 10.1038/nm.2285 21258338

[26] Lin PL Dietrich J Tan E Abalos RM Burgos J Bigbee C The multistage vaccine H56 boosts the effects of BCG to protect cynomolgus macaques against active tuberculosis and reactivation of latent *Mycobacterium tuberculosis* infection. J Clin Invest (2012) 122:303–14 10.1172/JCI46252 22133873 PMC3248283

[27] Biketov S Mukamolova GV Potapov V Gilenkov E Vostroknutova G Kell DB Culturability of *Mycobacterium tuberculosis* cells isolated from murine macrophages: a bacterial growth factor promotes recovery. FEMS Immunol Med Microbiol (2000) 29:233–40 10.1111/j.1574-695X.2000.tb01528.x 11118902

[28] Mukamolova GV Kaprelyants AS Young DI Young M Kell DB A bacterial cytokine. Proc Natl Acad Sci U S A (1998) 95:8916–21 10.1073/pnas.95.15.8916 9671779 PMC21177

[29] Davies AP Dhillon AP Young M Henderson B McHugh TD Gillespie SH Resuscitation-promoting factors are expressed in *Mycobacterium tuberculosis*-infected human tissue. Tuberculosis (Edinb) (2008) 88:462–8 10.1016/j.tube.2008.01.007 18440866

[30] Mukamolova GV Turapov OA Young DI Kaprelyants AS Kell DB Young M A family of autocrine growth factors in *Mycobacterium tuberculosis*. Mol Microbiol (2002) 46:623–35 10.1046/j.1365-2958.2002.03184.x 12410821

[31] Rachman H Strong M Ulrichs T Grode L Schuchhardt J Mollenkopf H Unique transcriptome signature of *Mycobacterium tuberculosis* in pulmonary tuberculosis. Infect Immun (2006) 74:1233–42 10.1128/IAI.74.2.1233-1242.2006 16428773 PMC1360294

[32] Mukamolova GV Turapov O Malkin J Woltmann G Barer MR Resuscitation-promoting factors reveal an occult population of tubercle bacilli in sputum. Am J Respir Crit Care Med (2010) 181:174–80 10.1164/rccm.200905-0661OC 19875686 PMC2809243

[33] Huang W Qi Y Diao Y Yang F Zha X Ren C Use of resuscitation-promoting factors proteins improves the sensitivity of culture-based tuberculosis testing in special samples. Am J Respir Crit Care Med (2014) 189(5):612–4 10.1164/rccm.201310-1899LE 24579840

[34] Gupta RK Srivastava BS Srivastava R Comparative expression analysis of rpf-like genes of *Mycobacterium tuberculosis* H37Rv under different physiological stress and growth conditions. Microbiology (2010) 156:2714–22 10.1099/mic.0.037622-0 20522500

[35] Yeremeev VV Kondratieva TK Rubakova EI Petrovskaya SN Kazarian KA Telkov MV Proteins of the Rpf family: immune cell reactivity and vaccination efficacy against tuberculosis in mice. Infect Immun (2003) 71:4789–94 10.1128/IAI.71.8.4789-4794.2003 12874362 PMC166051

[36] Geluk A van den Eeden SJ van Meijgaarden KE Dijkman K Franken KL Ottenhoff TH A multistage-polyepitope vaccine protects against *Mycobacterium tuberculosis* infection in HLA-DR3 transgenic mice. Vaccine (2012) 30(52):7513–21 10.1016/j.vaccine.2012.10.045 23103299

[37] Cho S Mehra V Thoma-Uszynski S Stenger S Serbina N Mazzaccaro RJ Antimicrobial activity of MHC class I-restricted CD8+ T cells in human tuberculosis. Proc Natl Acad Sci U S A (2000) 97:12210–15 10.1073/pnas.210391497 11035787 PMC17320

[38] Flynn JL Chan J Tuberculosis: latency and reactivation. Infect Immun (2001) 69:4195–201 10.1128/IAI.69.7.4195-4201.2001 11401954 PMC98451

[39] Lalvani A Brookes R Wilkinson RJ Malin AS Pathan AA Andersen P Human cytolytic and interferon gamma-secreting CD8+ T lymphocytes specific for *Mycobacterium tuberculosis*. Proc Natl Acad Sci U S A (1998) 95:270–5 10.1073/pnas.95.1.270 9419365 PMC18198

[40] Stenger S Mazzaccaro RJ Uyemura K Cho S Barnes PF Rosat JP Differential effects of cytolytic T cell subsets on intracellular infection. Science (1997) 276:1684–7 10.1126/science.276.5319.1684 9180075

[41] Ab BK Kiessling R Van Embden JD Thole JE Kumararatne DS Pisa P Induction of antigen-specific CD4+ HLA-DR-restricted cytotoxic T lymphocytes as well as nonspecific nonrestricted killer cells by the recombinant mycobacterial 65-kDa heat-shock protein. Eur J Immunol (1990) 20:369–77 10.1002/eji.1830200221 1690136

[42] Lewinsohn DA Winata E Swarbrick GM Tanner KE Cook MS Null MD Immunodominant tuberculosis CD8 antigens preferentially restricted by HLA-B. PLoS Pathog (2007) 3:1240–9 10.1371/journal.ppat.0030127 17892322 PMC2323292

[43] Smith SM Brookes R Klein MR Malin AS Lukey PT King AS Human CD8+ CTL specific for the mycobacterial major secreted antigen 85A. J Immunol (2000) 165:7088–95 10.4049/jimmunol.165.12.7088 11120838

[44] Smith SM Klein MR Malin AS Sillah J McAdam KP Dockrell HM Decreased IFN-gamma and increased IL-4 production by human CD8(+) T cells in response to *Mycobacterium tuberculosis* in tuberculosis patients. Tuberculosis (Edinb) (2002) 82:7–13 10.1054/tube.2001.0317 11914057

[45] Caccamo N Guggino G Meraviglia S Gelsomino G Di CP Titone L Analysis of *Mycobacterium tuberculosis*-specific CD8 T-cells in patients with active tuberculosis and in individuals with latent infection. PLoS One (2009) 4:e5528 10.1371/journal.pone.0005528 19436760 PMC2678250

[46] Lewinsohn DM Swarbrick GM Cansler ME Null MD Rajaraman V Frieder MM Human CD8 T Cell antigens/epitopes identified by a proteomic peptide library. PLoS One (2013) 8:e67016 10.1371/journal.pone.0067016 23805289 PMC3689843

[47] Tang ST van Meijgaarden KE Caccamo N Guggino G Klein MR van Weeren P Genome-based in silico identification of new *Mycobacterium tuberculosis* antigens activating polyfunctional CD8+ T cells in human tuberculosis. J Immunol (2011) 186:1068–80 10.4049/jimmunol.1002212 21169544

[48] Rodenko B Toebes M Hadrup SR van Esch WJ Molenaar AM Schumacher TN Generation of peptide-MHC class I complexes through UV-mediated ligand exchange. Nat Protoc (2006) 1:1120–32 10.1038/nprot.2006.121 17406393

[49] Lindestam Arlehamn CS Gerasimova A Mele F Henderson R Swann J Greenbaum JA Memory T cells in latent *Mycobacterium tuberculosis* infection are directed against three antigenic islands and largely contained in a CXCR3+CCR6+ Th1 subset. PLoS Pathog (2013) 9:e1003130 10.1371/journal.ppat.1003130 23358848 PMC3554618

[50] Kaufmann SH How can immunology contribute to the control of tuberculosis? Nat Rev Immunol (2001) 1:20–30 10.1038/35095558 11905811

[51] Available from: http://hla.alleles.org/alleles/class1.html

[52] Mazzarino P Pietra G Vacca P Falco M Colau D Coulie P Identification of effector-memory CMV-specific T lymphocytes that kill CMV-infected target cells in an HLA-E-restricted fashion. Eur J Immunol (2005) 35:3240–7 10.1002/eji.200535343 16224817

[53] Pietra G Romagnani C Mazzarino P Falco M Millo E Moretta A HLA-E-restricted recognition of cytomegalovirus-derived peptides by human CD8+ cytolytic T lymphocytes. Proc Natl Acad Sci U S A (2003) 100:10896–901 10.1073/pnas.1834449100 12960383 PMC196899

[54] Salerno-Goncalves R Fernandez-Vina M Lewinsohn DM Sztein MB Identification of a human HLA-E-restricted CD8+ T cell subset in volunteers immunized with *Salmonella enterica* serovar typhi strain Ty21a typhoid vaccine. J Immunol (2004) 173:5852–62 10.4049/jimmunol.173.9.5852 15494539

[55] Strong RK Holmes MA Li P Braun L Lee N Geraghty DE HLA-E allelic variants. Correlating differential expression, peptide affinities, crystal structures, and thermal stabilities. J Biol Chem (2003) 278:5082–90 10.1074/jbc.M208268200 12411439

[56] Cohen GB Gandhi RT Davis DM Mandelboim O Chen BK Strominger JL The selective downregulation of class I major histocompatibility complex proteins by HIV-1 protects HIV-infected cells from NK cells. Immunity (1999) 10:661–71 10.1016/S1074-7613(00)80065-5 10403641

[57] Heinzel AS Grotzke JE Lines RA Lewinsohn DA McNabb AL Streblow DN HLA-E-dependent presentation of Mtb-derived antigen to human CD8+ T cells. J Exp Med (2002) 196:1473–81 10.1084/jem.20020609 12461082 PMC2194265

[58] Garcia P Llano M de Heredia AB Willberg CB Caparros E Aparicio P Human T cell receptor-mediated recognition of HLA-E. Eur J Immunol (2002) 32:936–44 10.1002/1521-4141(200204)32:4<936::AID-IMMU936>3.3.CO;2-D 11920559

[59] Pietra G Romagnani C Falco M Vitale M Castriconi R Pende D The analysis of the natural killer-like activity of human cytolytic T lymphocytes revealed HLA-E as a novel target for TCR alpha/beta-mediated recognition. Eur J Immunol (2001) 31:3687–93 10.1002/1521-4141(200112)31:12<3687::AID-IMMU3687>3.0.CO;2-C 11745389

[60] Sarantopoulos S Lu L Cantor H Qa-1 restriction of CD8+ suppressor T cells. J Clin Invest (2004) 114:1218–21 10.1172/JCI200423152 15520850 PMC524234

[61] Joosten SA van Meijgaarden KE van Weeren PC Kazi F Geluk A Savage ND *Mycobacterium tuberculosis* peptides presented by HLA-E molecules are targets for human CD8 T-cells with cytotoxic as well as regulatory activity. PLoS Pathog (2010) 6:e1000782 10.1371/journal.ppat.1000782 20195504 PMC2829052

[62] Commandeur S van Meijgaarden KE Prins C Pichugin AV Dijkman K van den Eeden SJ An unbiased genome-wide *Mycobacterium tuberculosis* gene expression approach to discover antigens targeted by human T cells expressed during pulmonary infection. J Immunol (2013) 190:1659–71 10.4049/jimmunol.1201593 23319735

[63] Commandeur S van den Eeden SJF Dijkman K Clark SO van Meijgaarden KE Wilson L The *in vivo* expressed *Mycobacterium tuberculosis* (IVE-TB) antigen Rv2034 induces CD4+ T-cells that protect against pulmonary infection in HLA-DR transgenic mice and guinea pigs. Vaccine (2014) 32:3580–8 10.1016/j.vaccine.2014.05.005 24837764

